# *Sphingopyxis* sp. IM-1 Reduces Intact Microcystin-LR and Attenuates MC-LR-Associated Inflammatory Gene Expression in Hep3B Hepatocytes

**DOI:** 10.3390/toxins18080337

**Published:** 2026-08-01

**Authors:** Apurva Lad, Mudit Bhatia, Johnna A. Birbeck, Alex Kuang, Elliot Furr, Judy Westrick, Youngwoo Seo, Dae-Wook Kang, David J. Kennedy, Steven T. Haller

**Affiliations:** 1 Department of Medicine, University of Toledo, Mail Stop 1040, Health Education Building, Toledo, OH 43614, USA; apurva.lad@utoledo.edu (A.L.); david.kennedy@utoledo.edu (D.J.K.); 2 Department of Civil and Environmental Engineering, University of Toledo, Mail Stop 307, 3006 Nitschke Hall, Toledo, OH 43606, USA; mudit.bhatia@rockets.utoledo.edu (M.B.); daewook.kang@utoledo.edu (D.-W.K.); 3 Lumigen Instrument Center, Department of Chemistry, Wayne State University, Detroit, MI 48202, USA; jbirbeck@chem.wayne.edu (J.A.B.); alex.z.k@wayne.edu (A.K.); efurr@wayne.edu (E.F.); judy.westrick@wayne.edu (J.W.); 4 Department of Chemical Engineering, University of Toledo, Mail Stop 307, 3048 Nitschke Hall, Toledo, OH 43606, USA

**Keywords:** cyanotoxins, harmful algal blooms, hepatotoxicity, Hep3B cell line, microcystin-LR, probiotics, *Sphingopyxis* sp. IM1

## Abstract

Excessive cyanobacterial proliferation and toxin production increasingly threaten freshwater ecosystems, drinking water systems, and public health. Among cyanotoxins, microcystin-LR (MC-LR) is one of the most prevalent variants and is recognized for its hepatotoxicity, with evidence showing it can also alter gut microbiota composition. Previous studies, including our own, demonstrate that MC exposure can induce inflammation, oxidative stress, and changes in gene expression associated with immune responses, even at concentrations below guideline limits. This study investigated the protective effect of an MC-degrading bacterium, *Sphingopyxis* sp. IM1 (IM1) with a known enzymatic MC degradation pathway, against MC-LR-induced hepatotoxicity under in vitro conditions. Human Hep3B hepatocytes were pretreated with varying ratios of IM1 bacteria and subsequently exposed to 9.95 ppm of MC-LR for 24 h. RT-qPCR analysis demonstrated that MC-LR exposure strongly increased the expression of the inflammatory markers *TNFα* and *TGF-β1*, whereas pretreatment with IM1 significantly attenuated these responses. Mass spectrometric analysis of cell pellets and spent culture media demonstrated reduced concentrations of intact MC-LR in IM1-pretreated samples compared to MC-LR-only controls, accompanied by increased detection of tetrapeptide degradation products generated during *mlr*-mediated MC degradation. These results demonstrate that IM1-mediated degradation of MC-LR attenuates toxin-induced hepatotoxic and inflammatory responses, highlighting the potential of a novel microbial-based therapeutic approach to mitigate MC-induced toxicity.

## 1. Introduction

Cyanobacterial harmful algal blooms (cHABs) have become increasingly prevalent in freshwater systems, driven by escalating global temperatures and nutrient enrichment, leading to contamination of aquatic environments through the production of taste- and odor-related compounds and cyanotoxins [1,2,3,4]. Among cyanotoxins, microcystins (MCs) are the most prevalent in freshwater environments and are produced by various cyanobacterial genera, including *Microcystis*, *Planktothrix*, *Dolichospermum*, and *Nostoc* [5].

MCs are cyclic heptapeptides comprising two variable L-amino acids, resulting in more than 300 identified congeners [5,6]. Among these, MC-LR is the most extensively studied due to its environmental prevalence and high toxicity [3]. Produced via non-ribosomal pathways, MCs are potent hepatotoxins that adversely affect both animals and humans [7,8,9]. Acute exposure in humans can cause liver injury along with symptoms like nausea and vomiting, whereas chronic exposure is associated with liver lesions and an increased risk of hepatic cancer; in animals, high-level exposure can be fatal [10,11].

MC exposure occurs primarily through the ingestion of contaminated drinking water and food, although dermal contact during recreational activities and inhalation of aerosolized toxins can also contribute [12,13]. Following uptake, MCs are transported into cells via organic anion-transporting polypeptides (OATPs), particularly OATP1B1 and OATP1B3 [14,15]. Once inside the cell, MCs inhibit protein phosphatases 1 (PP1) and 2A (PP2A) by blocking the catalytic site of the phosphatases, resulting in their inhibition [8,16]. This inhibition leads to abnormal protein hyperphosphorylation, which further triggers a cascade of adverse cellular effects, including cytoskeletal disruption, oxidative stress, endoplasmic reticulum dysfunction, DNA damage, apoptosis, and inflammation mediated through the MAPK and NF-κB signaling pathways [17,18,19,20,21]. Although the liver is the primary target organ, studies have also documented MC-induced toxicity in the kidneys [22]. Due to its toxicity, the World Health Organization (WHO) established drinking water guideline values (GVs) for MC-LR in 2020, setting the long-term GV at 1 µg/L and the short-term GV at 12 µg/L, with local governing agencies implementing more stringent GVs to protect children and immunocompromised individuals [2,23,24].

MCs exhibit high stability in natural environments, and abiotic degradation only occurs under extreme conditions, such as very low pH, sonication, or H_2_O_2_-assisted UV irradiation [25,26]. Consequently, biological degradation by indigenous bacteria has gained attention as a promising remediation strategy because of its substrate specificity, cost-effectiveness, and limited formation of harmful byproducts [27,28,29,30,31,32]. MC-degrading bacterial strains have been isolated from cHAB-impacted waters and include members of the genera *Paucibacter*, *Novosphingobium*, *Sphingomonas*, and *Sphingopyxis* [33]. These strains are generally categorized based on the presence or absence of the *mlr* gene cluster, which encodes enzymes responsible for the stepwise biodegradation of MC-LR [34]. A study by Ding et al. demonstrated that the *mlr*-mediated MC biodegradation pathway ultimately produces a tetrapeptide byproduct containing the Adda–Glu–Mdha–Ala fragment containing seven amino acids [35].

Since their discovery, MC-degrading bacteria have been studied for applications in contaminated water remediation through bioaugmentation and natural enrichment, as well as within drinking water treatment systems, where they contribute to MC removal in biofilm communities on biological activated carbon (BAC) filters [30,36]. Although drinking water treatment utilities meet the stringent guidelines, trace MCs often persist in finished drinking water, albeit below regulatory thresholds. Importantly, growing evidence shows that MC-LR exposure at sub-threshold levels can still trigger hepatotoxic effects, including inflammation, oxidative stress, and dysregulation of immune-related genes [37].

The use of MC-degrading bacteria as a probiotic supplement could be a potential solution to the problem. Through the gut–liver axis, a bidirectional communication network linking the intestinal microbiota and hepatic function, probiotic-mediated degradation of MCs could reduce toxin bioavailability, thereby improving liver health and limiting inflammation and toxicity. Previous studies investigating MC-induced inflammation in renal and colorectal tissues have employed bacteria from the *Sphingopyxis* and *Lactobacillus* genera to alleviate inflammatory responses [38,39]. However, these approaches primarily targeted the physiological consequences of exposure and did not directly reduce MC concentrations, effectively treating symptoms rather than the underlying cause. Although mammalian cells possess some capacity to metabolize MCs, the use of targeted, rapidly degrading MC-degrading bacteria as probiotics could enhance toxin removal before absorption and accumulation, potentially reducing the long-term health impacts associated with chronic MC exposure. For example, probiotics from the *Sphingopyxis* genus have demonstrated mycotoxin-degrading capacity, with proposed applications as feed and fodder additives due to their presence in the gut microbiome of various animals [40,41,42].

Although bacterial degradation of MC has been vastly studied, most existing research focuses on environmental remediation rather than therapeutic application, with limited investigation into the in vitro efficacy of hepatoprotection. This study aims to address these gaps by investigating the potential of *Sphingopyxis*-IM1 (referred to as IM1), an *mlr*+ MC-degrader strain, as a probiotic intervention for MC-LR degradation and hepatotoxicity and inflammation reduction in Hep3B cells, a human hepatocellular carcinoma cell line. We hypothesize that targeted administration of this MC-degrading probiotic strain will effectively reduce MC concentrations while mitigating hepatic damage in exposed organisms.

The findings of this research provide valuable insights for developing novel microbiome-based interventions against environmental toxin exposure and offer a promising strategy for populations at risk of MC-induced liver diseases. By harnessing the inherent capabilities of probiotic bacteria to neutralize toxic compounds before they reach the liver, we may establish a new paradigm in preventive and therapeutic approaches to toxicant-induced hepatotoxicity.

## 2. Results

### 2.1. Treatment with IM1 Reduces MC-LR-Induced Hepatotoxicity

**Figure 1:**
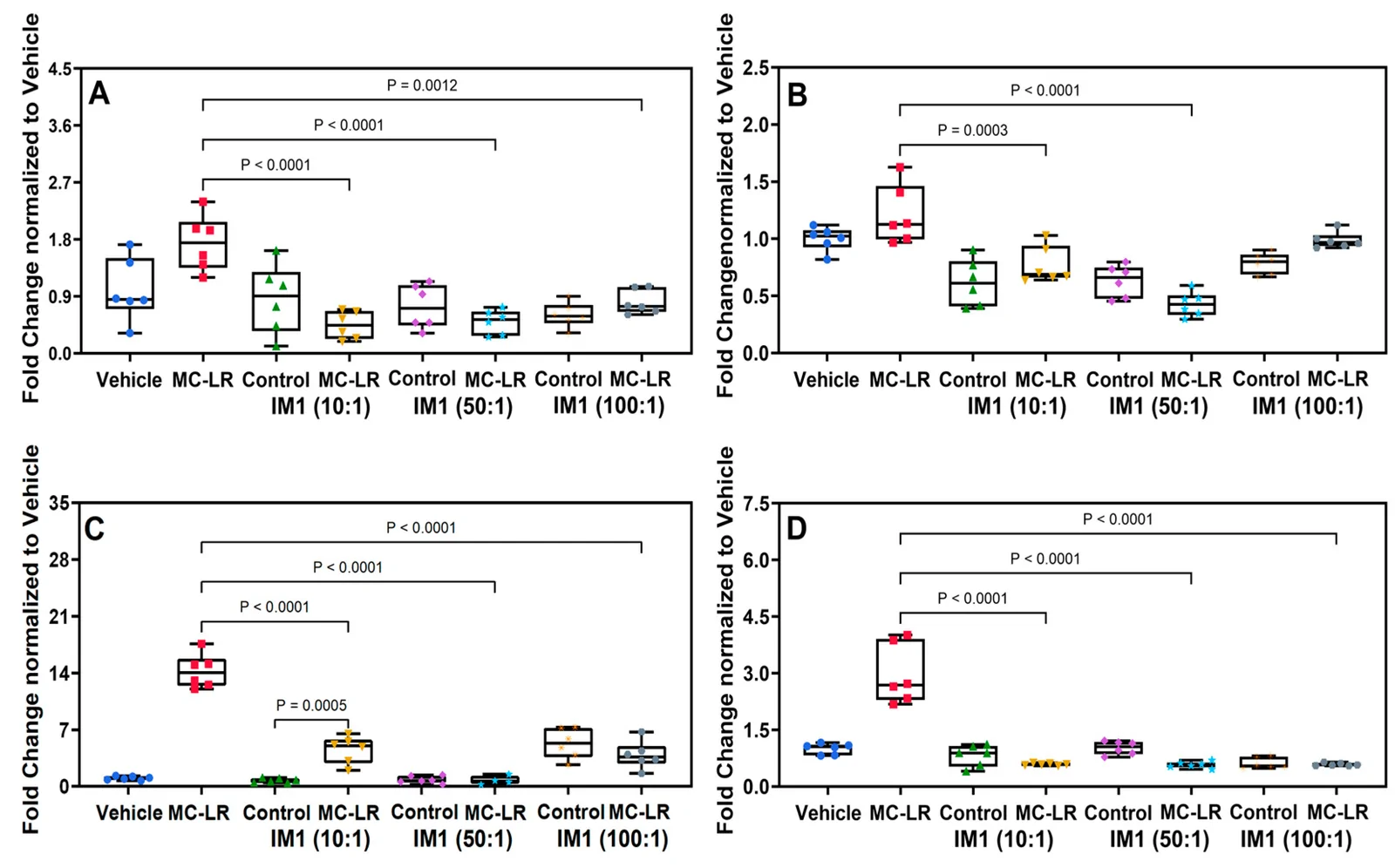
Pretreatment with cyanotoxin-degrading bacteria attenuates MC-LR-associated transcriptional responses. Quantitative PCR analysis of the exploratory hepatotoxicity-associated markers *OSMR* (**A**) and *SERPINE1/PAI-1* (**B**), and the inflammatory markers *TNFα* (**C**) and *TGF-β1* (**D**). MC-LR exposure strongly increased *TNFα* and *TGF-β1* expression, and these responses were significantly reduced by IM1 pretreatment. *OSMR* and *SERPINE1* exhibited smaller changes and are interpreted as exploratory markers. Statistical comparisons and corresponding *p*-values are shown in the individual panels.

RT-qPCR was used to evaluate two exploratory hepatotoxicity-associated markers, *OSMR* and *SERPINE1*, together with the inflammatory markers *TNFα* and *TGF-β1*. Using 18S normalization, MC-LR exposure increased *OSMR* expression to 1.7 ± 0.18-fold relative to vehicle-treated cells (1.0 ± 0.20) (Figure 1A). *SERPINE1* expression increased more modestly from 1.0 ± 0.04 in vehicle-treated cells to 1.21 ± 0.11 following MC-LR exposure, but this difference was not statistically significant (Figure 1B). Given the relatively small magnitude of these changes, *OSMR* and *SERPINE1* were interpreted as exploratory markers. The strongest MC-LR-associated transcriptional responses were observed for *TNFα* and *TGF-β1*.

Pre-treatment of hepatocytes with IM1 markedly reduced *OSMR* expression across all tested concentrations: 0.4 ± 0.2 (1:10), 0.5 ± 0.2 (1:50), and 0.8 ± 0.2-fold (1:100) (Figure 1A). No significant differences were observed between vehicle-treated cells and those pre-treated with IM1 alone, or IM1-pretreated cells subsequently exposed to MC-LR, indicating that IM1 by itself did not induce hepatotoxicity and effectively maintained *OSMR* expression at basal levels (Figure 1A).

MC-LR exposure produced a modest 1.21-fold increase in *SERPINE1* expression relative to vehicle-treated cells; however, this difference was not statistically significant. *SERPINE1* was therefore interpreted as an exploratory marker rather than as primary evidence of MC-LR-induced toxicity. However, IM1 pre-treatment affected expression in a concentration-dependent manner. At 1:10 (Hep3B:IM1), expression was reduced to 0.6 ± 0.2 (control) and 0.8 ± 0.2 (treatment), with a significant decrease compared to MC-LR alone for both control and treatment (Figure 1B). At 1:50, expression further decreased to 0.6 ± 0.1 (control) and 0.4 ± 0.1 (treatment), significantly lower than in both vehicle and MC-LR groups. At 1:100, a significant reduction was observed compared to MC-LR for control but not for treatment, with values of 0.8 ± 0.1 (control) and 0.9 ± 0.1 (treatment). Among controls, IM1 significantly lowered *SERPINE1* expression at 1:10 (*p* ≤ 0.01) and 1:50 (*p* ≤ 0.01) with respect to Vehicle (control) but not at 1:100. Similarly, in treatments, significant reductions relative to MC-LR were observed at 1:10 and 1:50, but not at 1:100 (Figure 1B).

Similarly, the inflammation markers *TNFα* and *TGF-β1* were strongly induced by MC-LR exposure, increasing by 14.2 ± 2.1 and 3.0 ± 0.8-fold, respectively, but were downregulated when cells were pre-treated with IM1 (Figure 1C,D). For *TNFα*, at 1:10 (Hep3B:IM1), expression in the control (0.7 ± 0.3) did not differ significantly from the vehicle (1.0 ± 0.2), while the treatment group with cells exposed to MC-LR and bacteria (4.5 ± 1.7) was significantly lower than MC-LR alone (Figure 1C). At 1:50, neither the control (0.8 ± 0.4) nor the treatment (0.7 ± 0.5) differed from the vehicle, and both were significantly reduced compared to MC-LR alone (Figure 1C). At 1:100, both the control (5.3 ± 1.9) and treatment (3.9 ± 1.7) showed significantly higher expression than the vehicle, but still significantly lower than MC-LR alone (Figure 1C). Notably, only the control at 1:100 exhibited a significant increase relative to the vehicle, whereas all three IM1 concentrations significantly reduced *TNFα* expression compared to MC-LR treatment (Figure 1C).

For the *TGF-β1* marker, IM1 treatment alone at any of the three concentrations (control) did not significantly alter gene expression compared to the vehicle (1.0 ± 0.1), with values of 0.8 ± 0.3, 1.0 ± 0.2, and 0.6 ± 0.1 for 10×, 50×, and 100× IM1, respectively (Figure 1D). In MC-LR–treated cells, co-treatment with IM1 at these concentrations reduced expression to 0.6 ± 0.03, 0.6 ± 0.1, and 0.6 ± 0.04, respectively, significantly lower than MC-LR alone and comparable to both the controls and the vehicle. These results indicate that the bacteria IM1, only at concentrations of 10× and 50× compared to hepatocytes, can potentially alleviate hepatotoxicity and inflammation caused by exposure to MC.

### 2.2. Mass Spectrometric Analysis Shows a Reduction in MC Levels on Treatment with IM1

**Figure 2:**
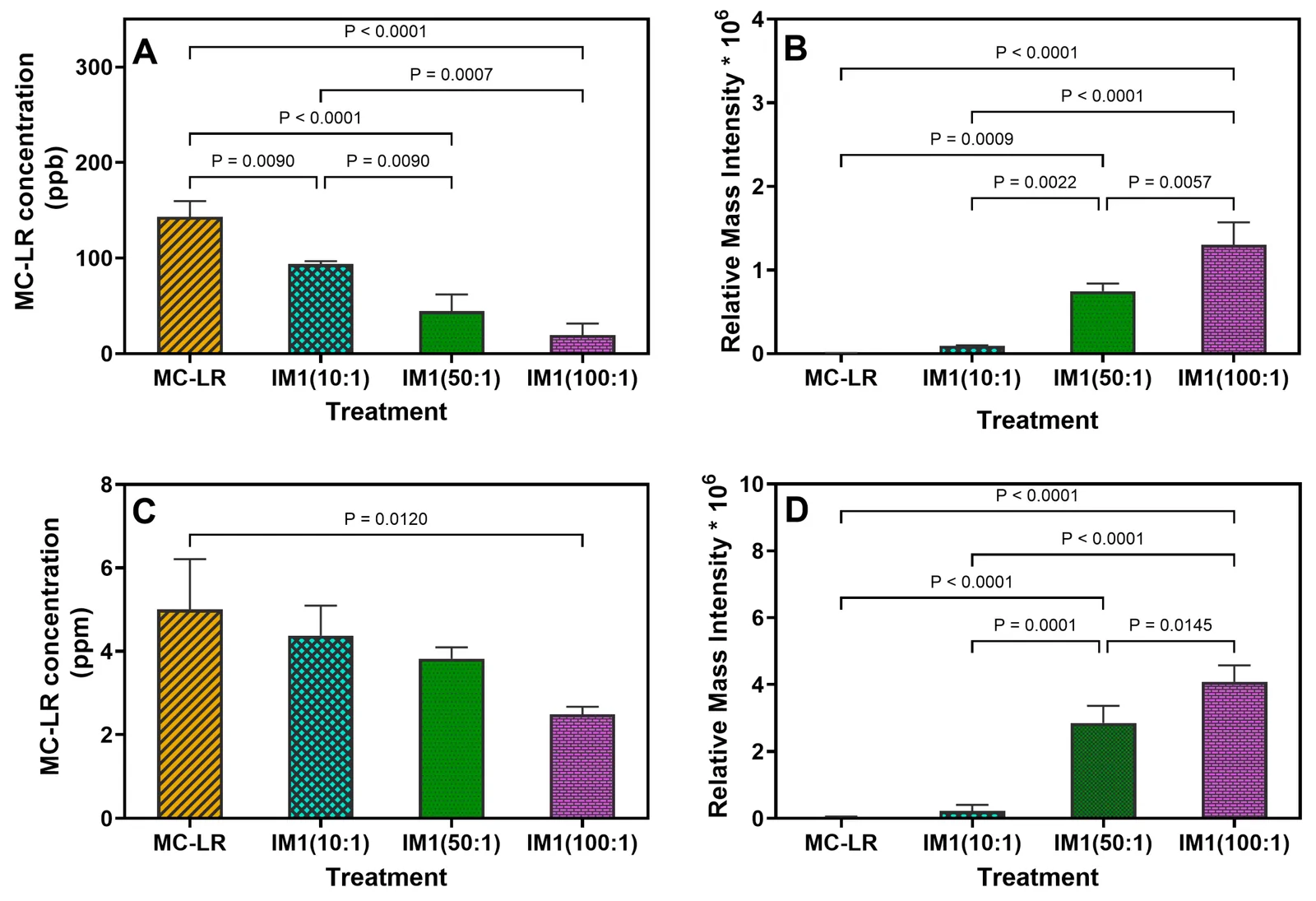
Pre-treatment with cyanotoxin-degrading bacteria significantly degrades MC-LR to tetrapeptide in a dose-dependent manner. Residual MC-LR (**A**,**C**) and tetrapeptide (**B**,**D**) concentrations in the cell pellet (**A**,**B**) and supernatant (**C**,**D**) were analyzed using LC-MS/MS.

To evaluate the role of MC-degrading bacteria in toxin removal and hepatocyte protection, a mass spectrometry-based method was used to quantify MC-LR and its degradation metabolite, the tetrapeptide, in both cell pellets and spent media. Each sample was initially spiked with 9.95 ppm MC-LR. In hepatocytes exposed to MC-LR alone, residual concentrations were 143.2 ± 16.4 ppb in the pellet (Figure 2A) and 5.0 ± 1.2 ppm in the spent media (Figure 2C), corresponding to 48.3% removal by hepatocytes. However, this clearance was associated with significant hepatocyte inflammation (Section 2.1). In contrast, IM1 supplementation produced a dose-dependent effect, with total degradation (pellet + spent media) increasing to 55.1%, 61.2%, and 74.8% at Hep3B:IM1 ratios of 1:10, 50, and 100, respectively (Figure 2A,C). IM1 augmentation resulted in an additional removal range of 6.8–26.5% beyond hepatocytes alone within 24 h which further resulted in a significant impact on toxicity, and inflammation reduction, demonstrating that IM1 enhances the MC-LR degradation efficiency of hepatocytes (Figure 1, Figure 2A,C).

Hepatocytes and IM1 cells degrade MC-LR via distinct pathways, producing different by-products. Hepatocyte metabolism converts MC-LR into the non-toxic MC-LR–cysteine conjugate, whereas IM1 degrades MC-LR through the *mlr*A pathway, generating a tetrapeptide intermediate that is further broken down. To determine the predominant degradation route in the presence and absence of IM1, we quantified tetrapeptide concentrations as an indicator of the *mlr*A-mediated pathway (Figure 2B,D).

**Figure 3:**
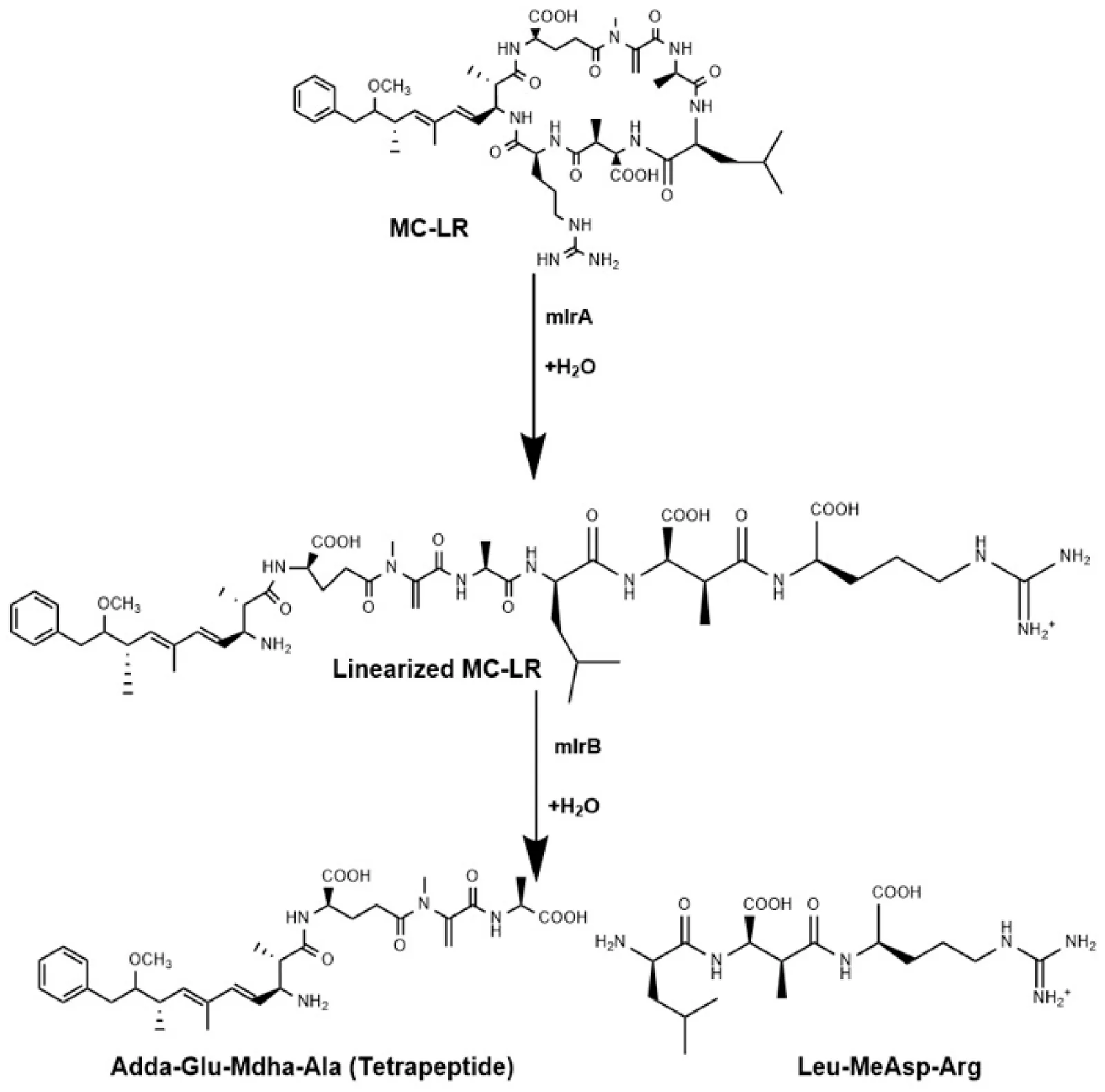
Schematic representation of MC-LR degradation via the mlrA-mediated pathway. The enzyme mlrA initiates the process by cleaving the cyclic structure of MC-LR to form a linearized intermediate, which is subsequently hydrolyzed by mlrB and mlrC into smaller peptide fragments, ultimately yielding a tetrapeptide containing the Adda–Glu–Mdha–Ala moiety.

Tetrapeptide accumulation followed a dose-dependent pattern, with higher concentrations detected in the supernatant compared to the cell pellet, in contrast to MC-LR, which was predominantly retained in the pellet. At a 1:10 Hep3B:IM1 ratio, the total tetrapeptide concentration (pellet + spent media) was 0.3 ± 0.2 × 10^6^ relative mass intensity, increasing to 3.6 ± 0.4 × 10^6^ relative mass intensity at 1:50 and 5.4 ± 0.7 × 10^6^ relative mass intensity at 1:100 (Figure 2B,D). Other metabolites generated during MC-LR degradation may include MC-LR–cysteine conjugates formed through hepatocyte-mediated detoxification pathway, as well as linearized MC-LR produced during IM1-mediated biodegradation both of which were not analyzed in this (Figure 3). These results suggest that IM1 complements hepatocyte metabolism by enhancing MC-LR degradation through the mlrA pathway in a dose-dependent fashion.

## 3. Discussion

Microcystins are one of the most common cyanotoxins documented worldwide and are predominant in most of the freshwater algal blooms in the United States [42]. In a survey of algal blooms in the Midwestern region of the United States, MC-LR and MC-RR emerged as the most prominent congeners [43,44,45]. For example, in Lake Erie, MC-LR and MC-RR account for approximately 90–95% of the total detected MC concentration [2]. Given the greater toxicity of MC-LR relative to MC-RR, together with previous studies demonstrating the ability of strain IM-1 to effectively degrade MC-LR, this congener was selected as the model toxin for the present study [30,46]. Till date, several genotypes of MC-degrading bacteria have been identified in aquatic environments and investigated for their potential to degrade toxins in drinking water treatment systems as part of bioremediation efforts [47,48,49,50,51,52]. In our previous study, we investigated the potential role of a mixture of cyanotoxin-degrading bacteria in ameliorating MC-LR-induced renal toxicity in vivo using a murine Balb/c model. We demonstrated that exposure to MC-LR significantly upregulated pathways involved in cellular respiration and metabolism and downregulated transcription pathways in the kidney. Pre-treatment with a mixture of five MC-degrading bacteria in drinking water before oral exposure to MC-LR effectively protected the kidneys. This pre-treatment was associated with upregulation of lipoprotein particle pathways and downregulation of respiration and ribosomal pathways, thus reversing the transcriptional profile induced by MC-LR exposure [53]. The concept of using probiotic bacteria to alleviate MC-induced toxicity is not novel, and various other studies have also investigated the potential role of different bacteria in ameliorating cyanotoxin-induced toxicity, particularly MC-LR, across a range of in vitro and in vivo models [54,55,56,57]. For example, in a study by Li et al., the authors conducted both in vivo and in vitro studies to determine the protective role of a different *Sphingopyxis* strain, namely, YF1, in mitigating MC-LR toxicity in the context of kidney injury. They also found that this *Sphingopyxis* strain can produce a bioactive compound—astaxanthin—which helped alleviate MC-LR-induced kidney injury via antioxidant and inflammatory pathways [39]. In another study by Yang et al., the authors demonstrated the potential of *Lactobacillus fermentum* to alleviate colorectal inflammation in C57BL/6J mice exposed to a low, subchronic dose of MC-LR [38]. However, MC-LR is a well-known hepatotoxin; the ability of the unique *Sphingopyxis* strain IM1 to mitigate this toxicity when co-cultured with hepatocytes remains unexplored. This study investigates the probiotic potential of IM1, its role in degrading MC-LR to reduce hepatotoxicity, and the optimum dose required to co-exist with hepatocytes under in vitro conditions without being pathogenic. Therefore, in this study, we used an established human hepatocyte cell line (Hep3B) that was exposed to an acute dose of MC-LR and treated with three different hepatocyte: bacteria cell ratios. Hepatotoxicity and inflammation were measured as fold changes in gene expression by quantitative PCR, while mass spectrometric analysis was used to quantify the toxin and its known bacterial breakdown product in cell pellets and spent media.

*SERPINE1*, which encodes plasminogen activator inhibitor-1, and OSMR have previously been associated with inflammatory signaling, tissue remodeling, and liver injury [58,59,60]. Up-regulation of this marker has also been associated particularly with liver damage as seen in fatty liver disease, bile duct ligation, hemorrhagic shock, and drug toxicity [61]. In our previous studies involving acute or chronic exposure to MC-LR, both in vitro [62] and in vivo [37,63], we have consistently observed considerable upregulation of *SERPINE1* expression. The cytokine Oncostatin M (OSM) is a member of the interleukin-6 (IL-6) family. The binding of OSM to its receptor, OSMR, activates the JAK-STAT signaling pathway, which is instrumental in downstream processes such as cell growth, apoptosis, and immune responses [64]. MC-LR is a well-known inducer of the PI3K/AKT pathway, which enhances cellular survival and pro-inflammatory cytokine biosynthesis, while concurrent JAK/STAT activation exacerbates inflammatory responses [65,66]. In the present study, 18S-normalized *OSMR* expression increased 1.7-fold following MC-LR exposure, whereas *SERPINE1* increased only 1.2-fold and did not differ significantly from the vehicle control. Because these changes were modest, particularly for *SERPINE1*, they are considered exploratory and should not be interpreted as definitive evidence of MC-LR-induced injury or IM1-mediated protection.

The more compelling transcriptional findings were the substantially larger increases in *TNFα* and *TGF-β1* following MC-LR exposure and their attenuation in cells pretreated with IM1. These inflammatory findings, together with the LC-MS/MS evidence of reduced intact MC-LR and increased detection of the *mlr*-associated degradation product, provide the primary support for the protective effect observed in this experimental system.

Previous studies have shown that MC-LR modulates immune responses and inflammatory cytokine expression in several animal models, including mice and fish [17,67,68]. A study by Yoshida et al. in mice given a single intraperitoneal (I.P.) injection of 60 µg/kg MC-LR and euthanized at various time points showed that *TNFα* gene expression was 2.3-fold higher than in controls [69]. This, along with our previous studies, is consistent with our observation that *TNFα* gene expression in Hep3B cells increased 14-fold upon exposure to MC-LR. However, the presence of bacteria reduced its expression, with a 1:50 dilution yielding the greatest reduction.

Another standard marker of inflammation is *TGF-β1*, which is more closely associated with liver fibrosis. A study by Chen et al. showed that exposure to MC-LR in a zebrafish model significantly increased *TGF-β1* expression, particularly in the intestine and gills [70]. Consistent with these observations, our study also showed significant upregulation of *TGF-β1* gene expression upon exposure to MC-LR, which was effectively reduced by treatment with cyanotoxin-degrading bacteria. Gene expression analysis demonstrated that exposure to MC-LR alone significantly elevates markers of inflammation and hepatotoxicity. While the 1:10 and 1:50 hepatocyte-to-IM1 ratios showed a clear dose-dependent response, the 1:100 ratio was slightly elevated and deviated from this trend. *Sphingopyxis* IM1 is a Gram-negative organism that contains lipopolysaccharides (LPS) within its cell wall. We hypothesize that a higher bacterial burden—and potentially a higher associated LPS burden—may itself stimulate inflammatory signaling and partially offset the benefit of additional MC-LR degradation. Because LPS concentration, cell viability, membrane integrity, and cytotoxicity were not directly measured, the present results cannot establish a formal maximum tolerated bacterial ratio or demonstrate that the 1:50 ratio is universally safe. However, the 1:50 ratio can be described as producing the most favorable response under the conditions tested, and direct safety and dose-ranging studies are required.

**Figure 4:**
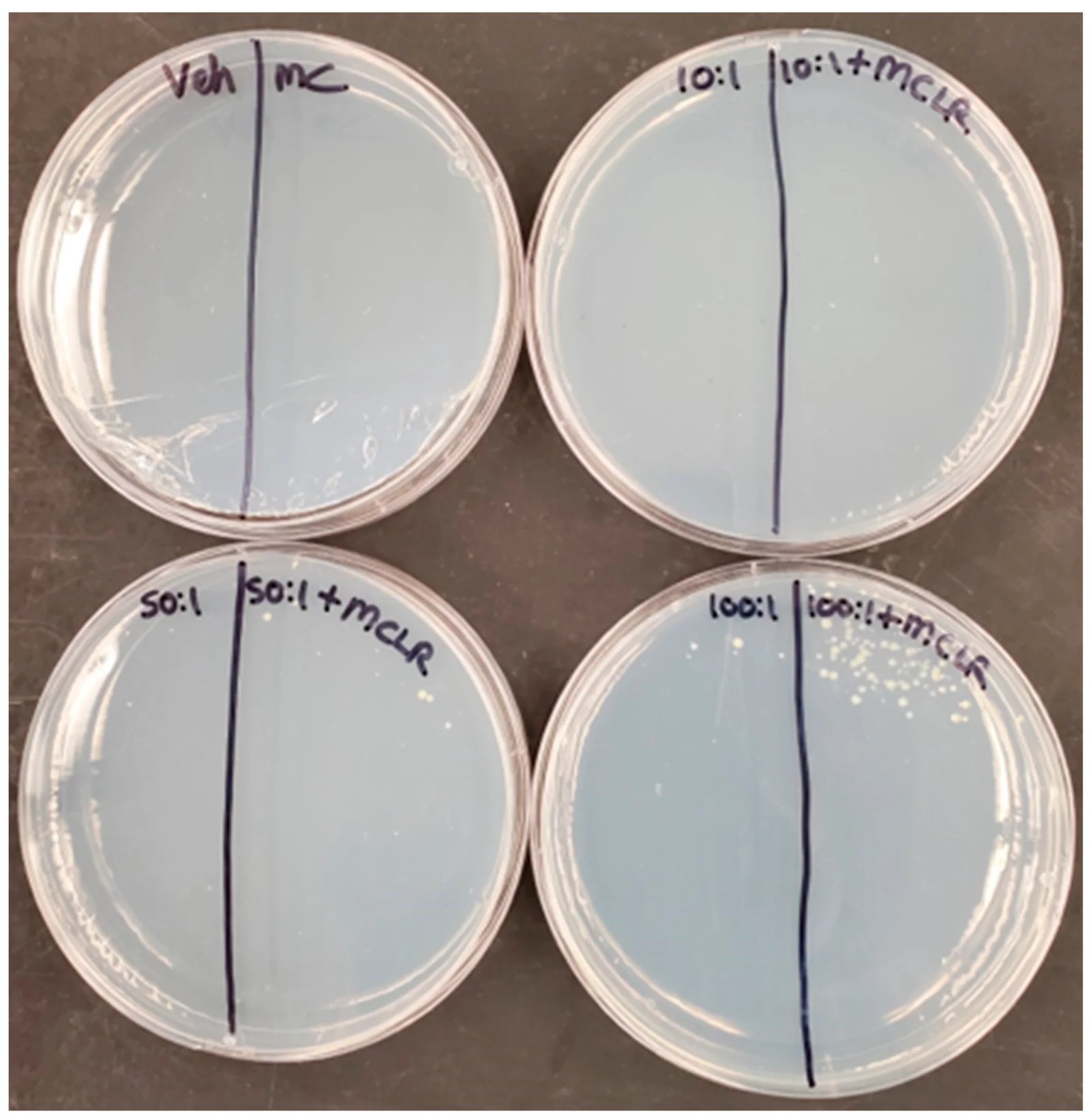
Representative image of the R2A plates streaked with the spent media from Hep3B hepatocytes and incubated at room temperature for 24 h. The observed colonies exhibited morphological characteristics consistent with the inoculated bacterial strain, and no evidence of contamination by extraneous microorganisms was detected.

As stated earlier, numerous studies have isolated and identified bacteria from around the world that are efficient at degrading MC [71,72]. These bacteria employ a variety of strategies to degrade the toxin, such as adsorption [38], the *mlr* gene cluster, and nutrient utilization [31]. Some others have also been shown to release a unique metabolite called astaxanthin, which has been shown to aid in the biodegradation of MC-LR, thus alleviating its toxicity [39]. It has been noted that the key products of *mlr*-assisted bacterial degradation of MC yield a linearized chain of heptapeptide, tetrapeptide, and ADDA, with some bacteria breaking it down further to individual amino acids [73]. In this study, we developed a mass spectrometric method to quantify and analyze the presence of intact MC-LR and the tetrapeptide by-product. To assess toxin breakdown, we quantified whole MC-LR and its *mlr*-degradation by-product, the tetrapeptide, in both the cell pellet and spent media. The dose-dependent decrease in total MC-LR, coupled with an inverse increase in tetrapeptide levels, confirms that IM1 actively degrades the toxin. The presence and purity of bacteria after treatment were also confirmed by streaking ~100 μL of the spent medium on R2A agar plates (Figure 4). No contaminating bacteria were observed. These observations indicate that the bacteria that assist in toxin degradation have potential as a therapeutic strategy to alleviate cyanotoxin-induced toxicity.

### Limitations

This study has several limitations that should be considered when interpreting the findings. Firstly, the investigation was limited to a single MC congener, MC-LR, based on its environmental and toxicological significance. MC-LR is the most prevalent microcystin congener detected in freshwater systems across Lake Erie and is recognized as one of the most potent hepatotoxic variants among cyanotoxins. Therefore, demonstrating bacterial-mediated detoxification of MC-LR provides an important proof of concept for the therapeutic potential of these MC-degrading microbes against cyanotoxin-induced hepatotoxicity. The efficacy of these cyanotoxin-degrading bacteria (IM1) against other clinically and environmentally relevant cyanotoxins remains to be established. Second, the 9.95 ppm MC-LR concentration was selected based on our previous dose-comparison studies in hepatocyte models, in which this concentration produced a reproducible molecular injury response over 24 h while permitting subsequent cellular and molecular analyses. Separate viability assays were not performed. The present experiment is an acute mechanistic proof-of-concept rather than a simulation of typical environmental exposure.

While our findings demonstrate promising hepatoprotective effects associated with MC-LR degradation, future investigations should evaluate the efficacy of IM1 against a broader spectrum of cyanotoxins, characterize the mechanisms underlying toxin degradation and host interactions, assess long-term safety and stability, and validate its effectiveness in diverse preclinical models. Such studies will be essential to fully elucidate the therapeutic potential and translational applicability of cyanotoxin-degrading bacteria for mitigating cyanotoxin-induced hepatotoxicity.

## 4. Conclusions

In conclusion, this study provides evidence that *Sphingopyxis* sp. IM1 can reduce intact MC-LR and attenuate MC-LR-associated inflammatory responses in Hep3B cells. MC-LR exposure strongly increased *TNFα* and *TGF-β1* expression, whereas pretreatment with IM1 significantly reduced these responses. *OSMR* and *SERPINE1* showed smaller changes and were therefore interpreted as exploratory markers rather than as primary evidence of hepatotoxicity or protection. LC-MS/MS analysis further demonstrated reduced intact MC-LR in IM1-treated samples, accompanied by increased detection of the *mlr*-associated tetrapeptide degradation product. Collectively, these findings support a relationship between IM1-mediated MC-LR degradation and attenuation of the cellular inflammatory response. To our knowledge, we are the first to study the role of *Sphingopyxis* sp. IM1 in ameliorating the hepatotoxic effects of MC-LR in vitro. These findings provide proof-of-concept that IM1-mediated MC-LR biodegradation can reduce intact toxin availability and attenuate MC-LR-associated transcriptional responses in Hep3B cells. Additional studies are required to establish responsible mechanisms, bacterial safety, gastrointestinal compatibility, efficacy against cyanotoxin mixtures, and translational feasibility.

## 5. Materials and Methods

### 5.1. Cell Line: Hep3B Cells

Hep3B hepatocytes, a human hepatocellular carcinoma cell line exhibiting epithelial morphology, were purchased from the American Type Culture Collection (ATCC) (Cat. No. HB-8064, ATCC, Manassas, VA, USA). The cells were grown in a complete media consisting of Eagle’s Minimal Essential Medium (EMEM—Base media) (Cat. No. 30-2003, ATCC, Manassas, VA, USA) supplemented with 10% Fetal Bovine Serum (FBS) (Cat. No. FBS-BBT, Rocky Mountain Biologicals, Missoula, MT, USA) and 1% penicillin-streptomycin solution (Cat. No. PSL01-100ML, Caisson Labs, Smithfield, UT, USA). The cells were grown and maintained in 75 cm^2^ tissue culture flasks (Cat. No. 229341, CELLTREAT, Ayer, MA, USA), incubated at 37 °C with 5% CO_2_. For the experiments, cells were grown and treated in 6-well plates (Cat. No. CC7682-7506, CytoOne, USA Scientific, Ocala, FL, USA).

### 5.2. Bacterial Culture Preparation

*Sphingopyxis* sp. IM1, a bacterium that degrades MC-LR via the *mlr* gene cluster, was obtained from IMDEA Water [32]. The bacteria were grown on Reasoner’s 2A (R2A) agar plates, and several colonies were transferred to 50 mL of sterilized R2A medium and incubated at room temperature at 110 rpm for 24 h. The culture was harvested at the late-exponential phase (O.D._585_ = 0.45) and centrifuged at 4500× *g* for 5 min. The pellet was washed three times using phosphate-buffered saline (PBS, pH 7.0) and resuspended in 5 mL of PBS. The culture O.D._585_ was adjusted to 0.1, corresponding to a concentration of 4 × 10^8^ CFU/mL, and added to the mammalian cell culture medium to achieve the desired treatment concentration.

### 5.3. Bacterial Treatment and MC-LR Exposure

Hep3B hepatocytes were used at Passage 4 and approximately 80% confluence. Before treatment, the FBS-containing medium was replaced with serum-free medium for 3 h to reduce serum-dependent background signaling. Lyophilized MC-LR (Cat. No. 10007188, Cayman Chemicals, Ann Arbor, MI, USA) was dissolved in UltraPure Distilled Water (Cat. No. 10977-015, Invitrogen, Waltham, MA, USA) to prepare a stock solution of 995 ppm, and cells were subsequently exposed to a final concentration of 9.95 ppm, equivalent to approximately 10 µM. This MC-LR concentration was determined based on our previous studies [62]. The ability of IM1 to mitigate MC-LR-induced hepatotoxicity was evaluated using three hepatocyte-to-bacteria ratios (1:10, 1:50, and 1:100) (Table 1), forming eight different experimental groups, including controls (Table 2).

**Figure 5:**
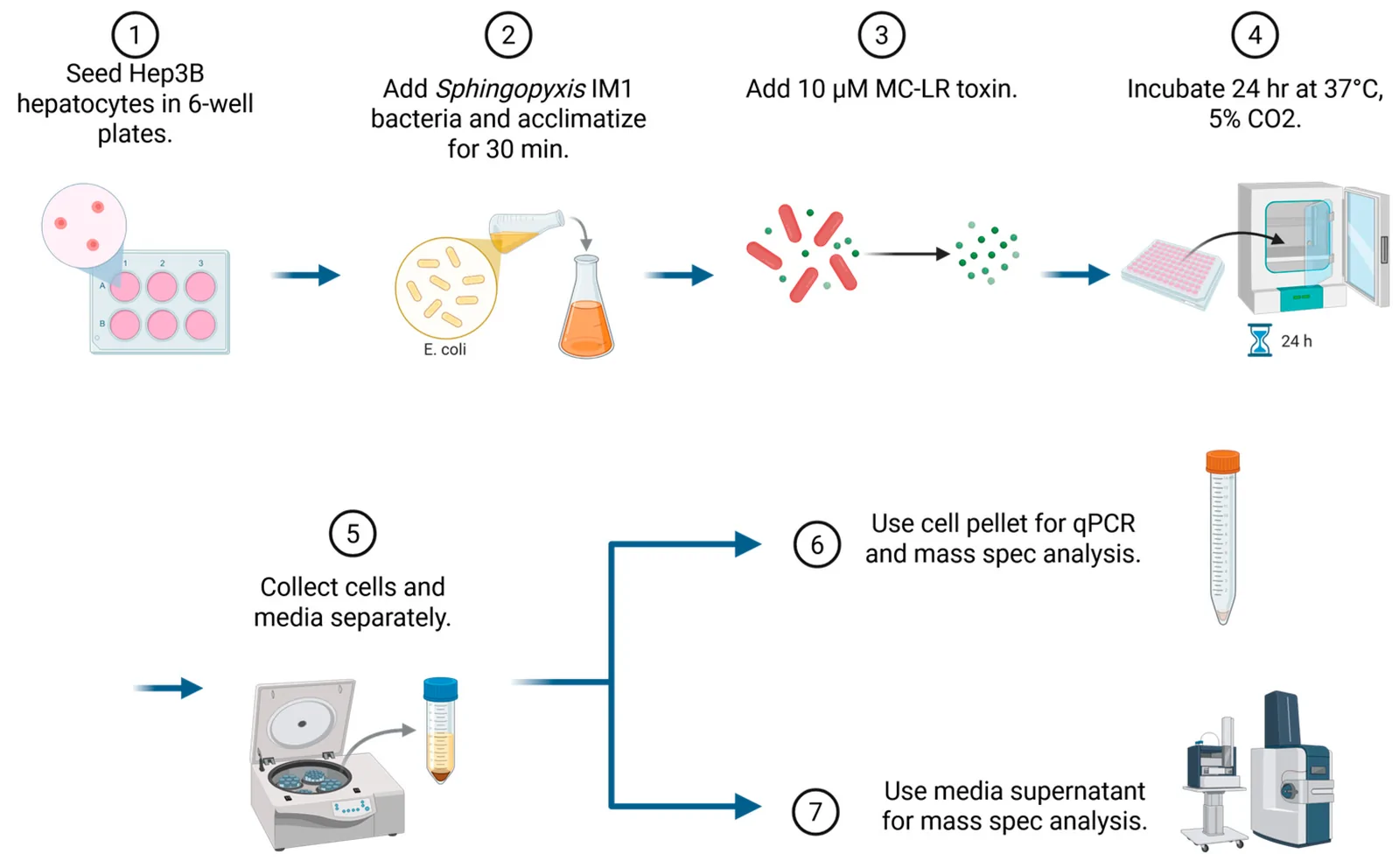
Schematic representation of the study design used to study the hepatoprotective effects of *Sphingopyxis* sp. IM1, the *mlr+* cyanotoxin-degrading bacteria, in an in vitro model using a human carcinoma hepatocyte cell line (Hep3B). The cells were pre-treated with the three hepatocyte: bacteria ratios for 30 min before being exposed to 10 µM of MC-LR for 24 h. After this, the cells and spent media were collected for further analysis. [Created in BioRender. Kennedy, D. (2026) https://BioRender.com/ldooyzx; accessed on 21 July 2026].

To determine the role of IM1 bacteria in MC degradation and hepatotoxicity alleviation, Hep3B cells in groups 3–8 were pre-treated with IM1 bacteria for 30 min (Table 2) before being exposed to 9.95 ppm MC-LR. The cells were then incubated for 24 h at 37 °C under 5% CO_2_ (Figure 5). All experiments were performed in triplicate with two independent experimental replicates.

### 5.4. RNA Extraction and Quantitative PCR Analysis

After 24 h of treatment, the spent media were collected and stored at −80 °C for mass spectrometric analysis. RNA from the treated cells was extracted using the RNeasy Plus Mini Kit (Cat. No. 74136, Qiagen, Germantown, MD, USA) according to the manufacturer’s protocol. Around 500 ng of the extracted RNA was used to synthesize cDNA using Qiagen’s RT^2^ First Strand Kit (Cat. No. 330401, Qiagen, Germantown, MD, USA). An automated liquid-handling workflow system, QIAgility, was used to load qPCR samples and reagents. qPCR was performed using a Qiagen 2-plex Rotor-Gene Q thermo-cycler. 18S rRNA (Cat. No. 4319413E, Thermo Fisher Scientific, Waltham, MA, USA) was used as a housekeeping gene for normalization. The following TaqMan primers obtained from Thermo Fisher Scientific, Waltham, MA, USA were used to assess hepatotoxicity [*SERPINE1* (Hs00167155_m1) and *OSMR* (Hs00384276_m1)] and inflammation [*TNFα* (Hs174128_m1) and *TGF-β1* (Hs00998133_m1)]. The cycle threshold values obtained in the process were used to calculate the fold change in gene expression levels.

### 5.5. Mass Spectrometric Analysis

Optima LC–MS grade solvents such as water, acetonitrile, methanol, acetic acid, and formic acid were purchased from Fisher Scientific (Tewksbury, MA, USA). MC-LR was purchased from Enzo Life Sciences, Inc. (Farmingdale, NY, USA), and the surrogate C2D5 MC-LR was purchased from Cambridge Isotope Laboratories, Inc. (Tewksbury, MA, USA).

The cells and spent media samples were stored at −80 °C. Cell pellets were resuspended in PBS and rinsed three times with 1 mL of PBS. After the spent media was removed, 0.5 mL of water was added, and the samples were lyophilized. 1.5 mL of water was then added to the samples, which were vortexed and centrifuged. The spent media was transferred to a 2 mL LC vial for analysis.

A high-throughput online concentration of LC-MS/MS was used to determine the concentrations of MC-LR and the tetrapeptide as described in Birbeck et al. [74]. Briefly, using a Thermo Scientific TSQ Altis™ 245 triple quadrupole mass spectrometer (Thermo Scientific, Waltham, MA, USA) with an EQuan 246 MAX Plus™ system, 1 mL of sample was injected onto a loading column (Thermo Scientific 247 Hypersil GOLD aQ 2.1 × 20 mm, 12 μm particle size) using an HTC PAL autosampler (CTC 248 Analytics, Zwingen, Switzerland). A Thermo Accucore aQ, 50 × 249 2.1 mm, 2.6 μm particle size analytical column, kept at a stable temperature of 35 °C, was used for gradient analysis. Mass spectrometric analysis was performed using a positive electrospray ionization source (ESI) 251 mode. Quantitation data results were obtained using TraceFinder™ EFS 4.1.

Samples were analyzed using a Thermo Scientific TSQ Quantiva™ triple quadrupole mass spectrometer coupled to an UltiMate™ 3000 ultra-high-performance liquid chromatography (UHPLC) system (Thermo Fisher Scientific, Waltham, MA, USA). Microcystin-LR (MC-LR) standards used throughout the study served as calibration and quality control standards for all mass spectrometric analyses. Electrospray ionization (ESI) was operated in positive ion mode, and quantitative analysis was performed using selected reaction monitoring (SRM). SRM transitions were monitored for the precursor ions of MC-LR (*m*/*z* 995) and the tetrapeptide metabolite (*m*/*z* 615), each producing the characteristic product ion at *m*/*z* 135.

Spent Media Culture: Chromatographic separation was performed on a Thermo Scientific Hypersil Gold column (2.1 × 50 mm, 1.9 μm) maintained at 35 °C at a flow rate of 0.35 mL min^−1^ [75]. The mobile phases consisted of (A) water containing 0.1% formic acid and (B) acetonitrile containing 0.1% formic acid. The gradient program began at 35% B and was held for 1.0 min, followed by a linear increase to 60% B over 1.0–4.75 min. The column was then washed with 98% B from 4.76 to 6.50 min before returning to the initial conditions (35% B) at 6.51 min and equilibrating until 9.50 min. The injection volume was 10 μL.

Quantification was performed using an external calibration curve prepared with MC-LR standards over the concentration range of 0.05–50 μg L^−1^. Samples with concentrations exceeding the upper calibration limit were diluted appropriately and reanalyzed. The method limit of detection (LOD) and limit of quantification (LOQ) for MC-LR were 0.05 and 0.10 μg L^−1^, respectively.

The mass spectrometer was operated with electrospray ionization in positive mode. Source parameters were as follows: spray voltage, 3.5 kV; ion transfer tube temperature, 260 °C; vaporizer temperature, 375 °C; sheath gas, 25 arbitrary units; auxiliary gas, 15 arbitrary units; and sweep gas, 1 arbitrary unit.

Cell Pellet: A 1.0 mL sample was loaded onto a Thermo Scientific Hypersil GOLD aQ trapping column (2.1 × 20 mm, 12 μm) using a TriPlus RSH autosampler at a flow rate of 1.5 mL min^−1^ with water containing 0.1% formic acid [74]. After a 1.0 min loading period, retained analytes were transferred onto an Accucore™ aQ analytical column (50 × 2.1 mm, 2.6 μm) for chromatographic separation. The mobile phases consisted of (A) water containing 0.1% formic acid and (B) acetonitrile containing 0.1% formic acid. Separation was performed at a flow rate of 0.60 mL min^−1^ using the following gradient: 24% B held for 0.7 min, increased to 26% B from 0.7 to 1.7 min, followed by a linear increase to 50% B from 1.7 to 5.5 min. The column was then washed with 98% B from 5.51 to 6.50 min at a flow rate of 1.0 mL min^−1^ before returning to the initial mobile phase composition for re-equilibration. The total run time was 8.5 min. The injection volume was 1.0 mL, and the column oven was maintained at 35 °C.

Quantification was performed using an external calibration curve prepared with MC-LR standards over the concentration range of 0.005–1000 μg L^−1^. Samples exceeding the upper calibration limit were diluted appropriately and reanalyzed. The method limit of detection (LOD) and limit of quantification (LOQ) for MC-LR were 0.005 and 0.010 μg L^−1^, respectively.

The mass spectrometer was operated with electrospray ionization (ESI) in positive ion mode. Source parameters were as follows: spray voltage, 3.5 kV; ion transfer tube temperature, 356 °C; vaporizer temperature, 420 °C; sheath gas, 52 arbitrary units; auxiliary gas, 16 arbitrary units; sweep gas, 2 arbitrary units; and collision gas pressure, 1.5 mTorr.

### 5.6. Statistical Analysis

Statistical analysis was performed using GraphPad PRISM 7 software (San Diego, CA, USA). A comparison was made between different experimental groups using Analysis of Variance (ANOVA). All data are presented as Mean ± Standard Error of Mean (S.E.M.), and a *p*-value of <0.05 was considered statistically significant.

## Figures and Tables

**Table 1:** Depicts the Hep3B: IM1 ratios and final concentrations/well chosen for the study.

Hep3B:IM1 Ratio	Hep3B Cells/Well	IM1 CFU/Well
1:10	9.5 × 10^5^	9.5 × 10^6^
1:50	9.5 × 10^5^	4.83 × 10^7^
1:100	9.5 × 10^5^	9.5 × 10^7^

**Table 2:** Depicts the different treatment groups and controls used in this study.

Group #	Group Name	Treatment
1	Vehicle (Negative Control)	Hep3B in EMEM and PBS
2	MC-LR (Positive Exposure Control)	Hep3B + 9.95 ppm MC-LR
3	IM1:10 Control	Hep3B + 10× IM1
4	IM1:10 MC-LR	Hep3B + 9.95 ppm MC-LR + 10× IM1
5	IM1:50 Control	Hep3B + 50× IM1
6	IM1:50 MC-LR	Hep3B + 9.95 ppm MC-LR + 50× IM1
7	IM1:100 Control	Hep3B + 100× IM1
8	IM1:100 MC-LR	Hep3B + 9.95 ppm MC-LR + 100× IM1

## Data Availability

The original contributions presented in this study are included in the article. Further inquiries can be directed to the corresponding authors.

## Abbreviations

The following abbreviations are used in this manuscript:

BAC	Biological Activated Carbon
cHABs	Cyanobacterial Harmful Algal Blooms
GV	Guideline Value
IM1	*Sphingopyxis* sp. IM1
LPS	Lipopolysaccharide
MC/MCs	Microcystin/Microcystins
MC-LR	Microcystin-LR
WHO	World Health Organization
